# Plasma fibulin-1 levels during pregnancy and delivery: a longitudinal observational study

**DOI:** 10.1186/s12884-021-04110-y

**Published:** 2021-09-17

**Authors:** Astrid Bakke Orvik, Malene Rohr Andersen, Lise Pedersen, Christian Ritz, Steen Stender, Pal Bela Szecsi

**Affiliations:** 1 Department of Gynecology and Obstetrics, Copenhagen University Hospital Rigshospitalet, Copenhagen, Denmark; 2 Department of Clinical Biochemistry, Herlev and Gentofte Hospital, Hellerup, Denmark; 3 Department of Clinical Biochemistry, Copenhagen University Hospital Holbaek, Holbaek, Denmark; 4 Department of Nutrition, Exercise and Sports, University of Copenhagen, Copenhagen, Denmark

**Keywords:** Fibulin, Humans, Plasma, Postpartum, Pre-eclampsia, Pregnancy, Preterm premature rupture of membranes

## Abstract

**Background:**

Fibulin-1 is an extracellular matrix protein expressed at high levels in the placenta. Elevated circulating fibulin-1 have been observed in women with severe pre-eclampsia, whereas low levels have been found in the fetal membranes, prior to membrane rupture. The aim of the study was primarily to evaluate plasma fibulin-1 during expected normal pregnancy and delivery, and secondarily to explore fibulin-1 levels in women developing pre-eclampsia or preterm premature rupture of fetal membranes (PPROM).

**Methods:**

From the historical longitudinal cohort originally consisting of 801 healthy Danish women with a singleton pregnancy, 128 women (632 samples) were selected. Of these, 107 women had normal pregnancies, nine experienced PPROM, and 12 pre-eclampsia. All samples were analyzed for fibulin-1, and levels were compared with blood donors. Differences in mean fibulin-1 between groups were estimated using a linear mixed model.

**Results:**

The mean concentration of fibulin-1 in 120 blood donors was 15.7 µg/mL, (25th-75th-percentiles, 12.3–18.2), with no significant difference in groups stratified by gender or age. Compared to baseline levels in week 12–20, fibulin-1 levels increased significantly from week 29–34 (estimated difference, 5.6 µg/mL; standard error, 1.7; *p* < 0.001) and 35–42 (12.5 µg/mL; 1.6; *p* < 0.001) and normalized after birth. The decrease at delivery tended to be more pronounced after elective (-7.0 µg/mL; 2.3; *p* = 0.002) and emergency (-5.6 µg/mL; 2.9; *p* = 0.05) cesarean section than after vaginal delivery (reference group). Women who developed PPROM had lower fibulin-1 levels throughout their pregnancies (-11.6 µg/mL; 4.2; *p* = 0.006). We did not observe a correlate between late pre-eclampsia and fibulin-1 (-0.2 µg/mL; 3.0; *p* = 0.9).

**Conclusions:**

Fibulin-1 was above non-pregnant levels at week 12 and increased significantly throughout pregnancy. We observed an association between low levels of fibulin-1 and PPROM. Further studies are needed to examine if fibulin-1 could serve as biomarker for the risk of PPROM. However, its role in late preeclampsia is doubtful.

**Trial registration:**

The study was conducted in accordance with the Declaration of Helsinki. The participants provided written informed consent, including storage for future use. The study was approved on July 18, 2005 by The Danish National Committee on Bioethics (No. KA 05065 and S-20,090,061) and the Danish Data Protection Agency.

**Supplementary Information:**

The online version contains supplementary material available at 10.1186/s12884-021-04110-y.

## Background

Fibulin-1 is a member of a family comprised of eight extracellular matrix glycoproteins that are characterized by a molecular weight of ≈ 100 kDa and a modular structure. The gene encoding fibulin-1 (FBLN1) is located on the long arm of human chromosome 22 (22q13.3), and the protein is detected in the extracellular matrix in many organs, often in relation to membranes, elastic fibers, and other connective tissue structures, suggesting a role in matrix remodeling [1, 2]. Fibulin-1 is found in plasma and has been detected in fetuses during early embryonic development [3]. Fibulin-1 mRNA and protein is expressed at high level in the placenta, according to tissue-specific oligonucleotide arrays using human and mouse samples [4]. In addition to roles in the differentiation of heart, skeletal, and neuronal structures in the early human embryo, fibulin-1 is associated with a variety of conditions [5, 6], including cancer, cardiovascular events, impaired kidney function [7], diabetes [7] and polycystic ovary syndrome [8]. Higher plasma levels of fibulin-1 have been observed in women with severe pre-eclampsia (PE), compared to healthy, gestational age-matched controls [9]. In addition, fibulin-1 and two other members of the fibulin family, have been found to be decreased in the para-cervical weak zone of the fetal membranes, prior to rupture [10].

To our knowledge, no study published to date has performed a longitudinal analysis of plasma fibulin-1 levels in healthy pregnant women. We hypothesized that plasma fibulin-1 levels vary with the gestational age and explored if the levels differ in women with normal, PE, and preterm premature rupture of membranes (PPROM) pregnancies [9–12]. In the present study, we report gestational age-specific plasma fibulin-1 concentrations in Caucasian Danish women during normal pregnancy, at delivery, and in the early postpartum period.

## Methods

### Study design and participants

**Fig. 1:**
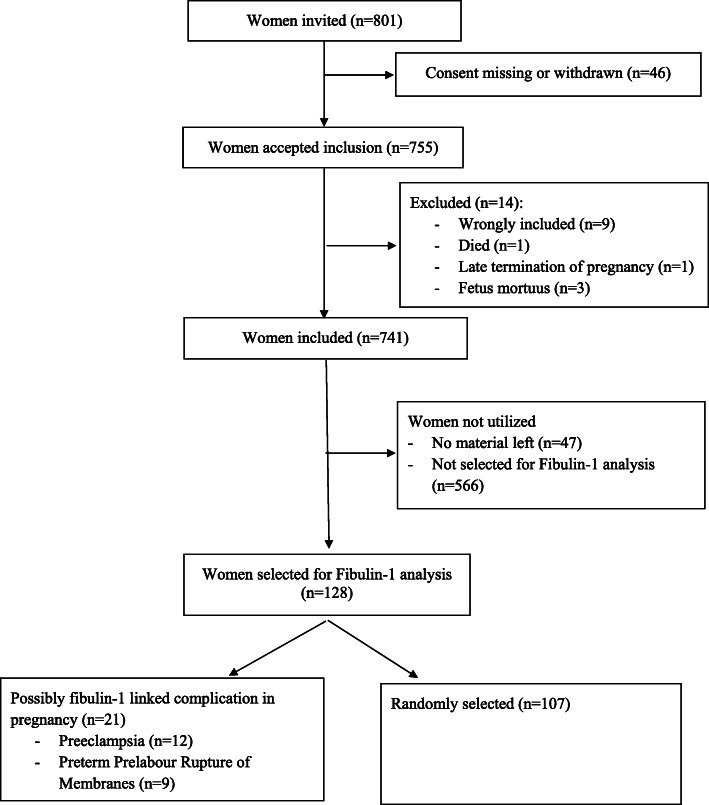
Consort flow chart

In the original cohort, 801 healthy Caucasian women aged greater than 18 years with an expected normal singleton pregnancy were recruited among 2147 women attending a first trimester screen (week 11 + 4 to 13 + 6) between June 2006 and October 2007 [13–18]. Clinical data were obtained from pregnancy charts and medical records. PE was defined as blood pressure above 140/90 mmHg debuting after week 20 + 0 and either proteinuria or signs of organ dysfunction. Early PE was defined as occurring before week 34 + 0, late PE after week 34 + 0. Severe PE was defined as blood pressure above 160/110, and/or subjective symptoms or laboratory findings consistent with severe organ dysfunction. PPROM was defined as rupture of membranes without uterine contractions before week 37 + 0. Preterm birth was defined as birth before week 37 + 0. Low birth weight was defined as a birth weight < 2500 g, regardless of gestational age. Postpartum hemorrhage was defined as blood loss of > 500 mL during a vaginal birth and > 1000 mL during cesarean section. Each woman was scheduled for seven blood collections at gestational weeks 13–20, 21–28, 29–34, and 35–42; at active labor or cesarean section; and on the first and second days after delivery. Blood samples were obtained and stored for future use. Due to financial restraint, only a subgroup of 128 women from the original cohort were selected for fibulin-1 quantification (Fig. 1). Of these, 107 women were randomly selected, as well as all available cases that developed PE (*n* = 12) or PPROM (*n* = 9).

### Reference range of Fibulin-1 levels in blood donors

Serum samples were obtained from a cohort of healthy adult Danish blood donors in August and September 2011 at Odense University Hospital, Denmark. One hundred twenty donors were included, with a target of 30 donors in each subgroup stratified by gender and greater than and less than 40 years of age (numbers obtained 62 females, 58 males; age range: 19–66 years) [19]. Informed consent was obtained, and the study was performed according to the Declaration of Helsinki. Immediately after sampling, all samples were aliquoted and stored at -80 °C until analysis. The reference range of fibulin-1 levels was established in 2015 using an immunoassay kit (CY-8094, Circulex, Medical & Biological Laboratories, Nagoya, Japan) with a manual setup on a Victor X5 Multiplate Reader (PerkinElmer, Waltham, MA) and verified in 2020 on 20 newly collected normal samples (from 4 men and 16 women, age range: 23–60 years) on the current platform according to the CLSI EP28-A3c guideline. No difference in serum and plasma fibulin-1 concentrations have been observed according to the kit manufacturer.

### Fibulin-1 analysis

Blood samples were collected and transported to the laboratory, where they were stored at -80 °C until use. Fibulin-1 levels were measured in ethylenediaminetetraacetic acid-treated plasma diluted 1:500 using the Circulex immunoassay kit on a Triturus 4-Plate platform (Grifols, Barcelona, Spain) according to the manufacturer’s instructions. The intra-assay and inter-assay coefficients of variation were < 8 % and < 12 % at a level of 10 µg/mL, respectively. The detection limit of the assay was 0.9 µg/mL, and serum samples were diluted in parallel to standard curve. The results were not available to the clinicians during the study.

### Statistical analysis

Data were analyzed using SPSS version 25 (IBM Corp, Armonk, NY, USA) and R software [20]. Differences in mean fibulin-1 levels between groups and associations with predictors were estimated using a linear mixed model. The model was fitted using the restricted maximum likelihood estimation, and included all predictors simultaneously, with the only exception when a continuous predictor was replaced with its dichotomized version for age and body mass index (BMI), ensuring complete adjustment for measured confounders. The following predictors (fixed effects) were simultaneously investigated: gestational period, PPROM, PE, mode of delivery, bleeding at delivery, birth weight, parity, smoking, BMI or BMI-group, and age or age-group. Participant-specific random intercept effects were included in the linear mixed model to capture the biological between-participant variation. Estimated mean differences and slope coefficients were reported. Based on the linear mixed models, post hoc pairwise comparisons using approximate t-tests were performed to quantify relevant differences. Differences were considered significant at *p* < 0.05. *P*-values were not adjusted for multiple comparisons due to the exploratory and hypothesis-generating nature of the study. Univariate and multivariate linear regression analyses were performed to investigate any correlations between fibulin-1 levels measured around time of birth with birthweight, bleeding at delivery and mode of delivery.

## Results

Fibulin-1 levels were measured in 632 samples from 128 of the 801 originally enrolled healthy pregnant Caucasian women. Five hundred forty-two of these samples were collected from 107 randomly selected women, 61 from 12 women with PE and 29 from 9 women with PPROM. Each woman contributed an average of 4.9 (range 1–8) samples. Five women attended their 1st trimester screening between week 12 + 2 to 12 + 6. The cohort had a mean (standard deviation) age of 32 (4.2) years, a BMI of 22 (2.7) kg/m^2^, a birth weight of 3442 (523) grams, and a gestational age at delivery of 39 + 5 (13) days.

Among the 107 randomly selected women, one woman was successfully administered tocolysis in week 29 + 5 due to premature contractions. None of the women experienced antepartum bleeding, glycosuria, isolated elevated blood pressure, isolated proteinuria, intrauterine growth restriction, or thrombosis in pregnancy. They all delivered at term: 24 by elective cesarean section (mainly due to maternal request and breech presentation), 12 by emergency cesarean section and the remaining 71 vaginally. Postpartum hemorrhage occurred in three women. Low birth weight occurred once (2450 g).

Among the 12 women with PE, one woman was treated with an anticoagulant in week 33 + 2 due to a suspicion of pulmonary embolism. None of the 12 women developed early PE. Two developed severe PE. All but one woman delivered at term, one delivered by elective cesarean section, two delivered by emergency cesarean section, and the remaining nine delivered vaginally. Postpartum hemorrhage occurred in two women, and a single child had low birth weight (1983 g).

Among the nine women with PPROM, all delivered vaginally and preterm (at gestational age 27 + 4 to 36 + 5). None experienced postpartum hemorrhaging. Low birth weight occurred in four infants (870, 1225, 1800, and 2042 g).

In blood donors, the mean concentration of fibulin-1 was 15.7 µg/mL, (SD 4.5, 25th -75th -percentiles, 12.3–18.2), with no significant difference in groups stratified by gender or age, and thus, a common reference interval was used.

**Fig. 2:**
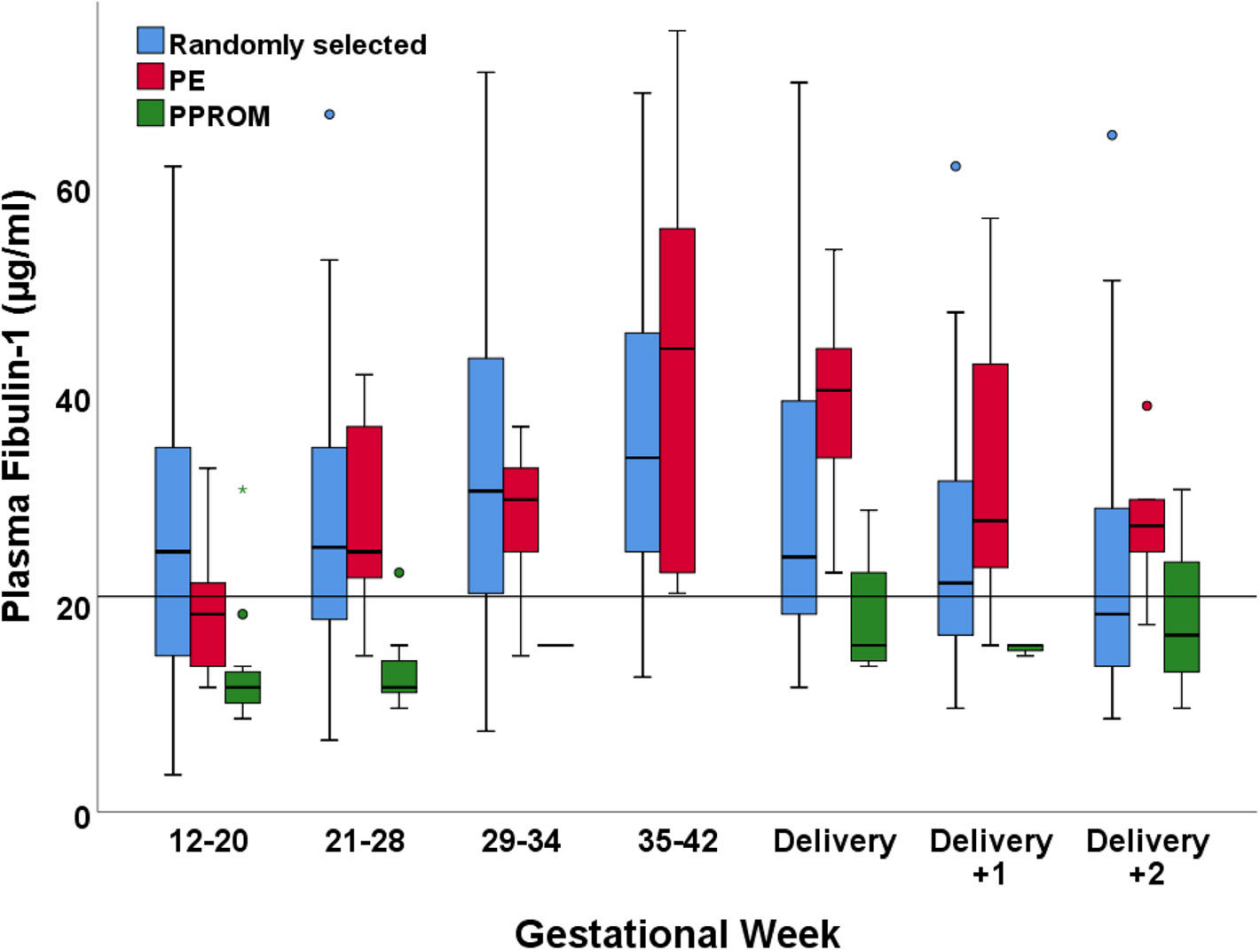
Gestational age-specific variation in plasma fibulin-1 levels during pregnancy. Plasma fibulin-1 concentrations in 632 samples from 128 healthy pregnant Caucasian women. The box plots represent the range of data from the 25^th^ to the 75^th^ percentiles, while the bar in the middle of each box plot represents the median value. The whiskers extending from the box represent the range of values, excluding outliers. Circles indicate outliers (1.5 x the interquartile range). The line represents the 90^th^ reference percentile in blood donors

Fibulin-1 concentrations in different demographic subgroups is shown in Table 1. The mean concentration of fibulin-1 in the pregnancy cohort was 27.4 (17.0-35.5) µg/mL. The lowest and highest concentrations measured were 3.6 µg/mL and 75.0 µg/mL, respectively. Among the randomly selected women, women with PE and women with PPROM, the mean concentrations of fibulin-1 were 27.8 (17.0–36.0) µg/mL, 29.1 (19.0–37.0) µg/mL, and 15.4 (11.0-16.5) µg/mL, respectively. Gestational age-specific plasma concentrations are shown in Fig. 2. Women who developed PPROM generally presented with low plasma fibulin-1 levels throughout their pregnancies. Women who developed late PE did not differ in plasma fibulin-1 levels compared to the randomly selected women.

**Table 1:** Characteristics of 128 healthy pregnant Caucasian women, 120 blood donors and their plasma Fibulin-1 concentrations

	Women		Samples		Fibulin-1, μg/mL	
	n	%	n	%	mean	SD
**Non-pregnant reference group**^**a**^	62F/58M		120		15.7	4.5
**Pregnant women overall**	128		632		27.4	13.2
**Age** (years)	128		632			
20-29	44	35	198	31	25.6	12.9
30-39	81	63	418	66	28.2	13.3
≥40	3	2	16	3	28.7	12.2
**Parity**	128		632			
0	51	40	255	40	25.1	12.8
1	59	46	289	46	28.2	13.4
2+	18	14	88	14	31.3	12.7
**Body mass index (kg/m**^**2**^**)**	127		628			
Underweight <18.5	5	4	22	4	18.7	10.0
Normal weight 18.5-24.9	98	77	491	78	27.8	13.1
Overweight 25.0-29.9	22	17	107	17	27.9	13.9
Obese 30.0-34.9	2	2	8	1	22.5	9.0
**Smoking habits**	128		632			
Non-smoker	107	84	531	84	28.2	13.7
Smoker	21	16	101	16	23.2	9.3
**Mode of conception**	128		632			
Spontaneous	126	98	625	98	27.5	13.2
In-vitro fertilization	1	1	3	1	16.3	1.1
Insemination	1	1	4	1	14.3	1.3
**Gestational weeks**	-		632			
12-20	66	-	93	15	23.4	12.2
21-28	90	-	121	19	26.4	11.4
29-34	59	-	60	10	32.8	14.7
35-42	50	-	63	10	36.0	14.3
Delivery	98	-	98	15	30.4	14.6
1^st^ day after delivery	101	-	101	16	25.2	10.9
2^nd^ day after delivery	96	-	96	15	22.7	10.8
**Pre-eclampsia**	128		632			
Not present	116	91	571	90	27.3	13.2
Present	12	9	61	10	29.1	13.1
**PPROM**	128		632			
Not present	119	93	603	95	28.0	13.1
Present	9	7	29	5	15.4	6.0
**Mode of delivery**	128		632			
Vaginal birth	88	69	396	63	28.5	13.1
Elective cesarean section	24	19	147	23	27.4	13.8
Emergency cesarean section	16	12	89	14	23.2	12.0
**Postpartum haemmorhage**^**b**^	128		632			
Not present	120	94	580	92	27.7	13.4
Present	8	6	52	8	23.4	9.4
**Birth weight (grams)**	126		621			
<2500	6	5	20	3	23.6	14.3
2500-2999	18	14	84	13	24.6	11.3
3000-3499	42	33	215	35	27.3	13.4
3500-3999	43	34	224	36	29.7	14.1
3999>	17	14	78	13	25.1	10.8

Fibulin-1 concentrations in different socio-demographic and clinical subgroups of the total study population. The data are presented as counts, percentages (%), or means and standard deviations (SD). ^a^Non-pregnant reference group consist of 62 female (F) and 58 male (M) blood donors. ^b^ Defined as >500 mL during vaginal delivery and >1000 mL during cesarean section

Notably, two women with non-spontaneous conceptions for whom 1st or 2nd trimester samples were not available, presented with low fibulin-1 levels in the 3rd trimester and at delivery (maximal value, 17.0 µg/mL).

**Fig. 3:**
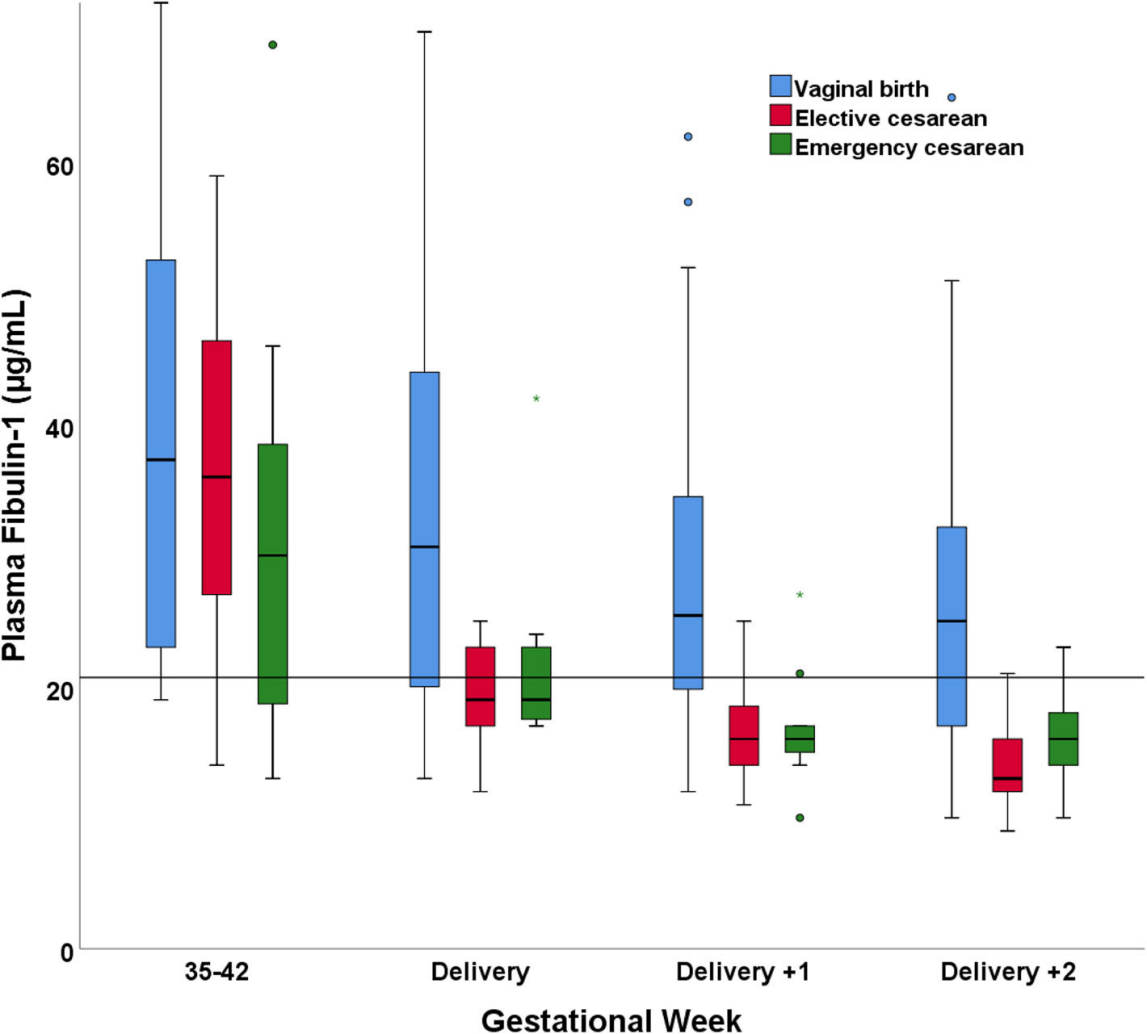
Fibulin-1 levels and mode of delivery. Plasma fibulin-1 concentrations in 3^rd^ trimester and around delivery measured in participants grouped according to the mode of delivery. The box plots represent the range of data from the 25^th^ to the 75^th^ percentiles, while the bar in the middle of each box plot represents the median value. The whiskers extending from the box represent the range of values excluding outliers. Circles indicate outliers. The line represents the 90^th^ reference percentile in blood donors

Compared to baseline levels measured in the early 2nd trimester and after adjusting for other variables, a significant increase in fibulin-1 concentrations was observed from week 29 throughout delivery (Table 2). An observed and predicted peak was observed in weeks 35–42. The level of fibulin-1 was significantly different between weeks 21–28 and 29–34 (*p* = 0.003), weeks 29–34 and 35–42 (*p* < 0.001), weeks 35–42 and delivery (*p* < 0.001), and delivery and the 1st day after delivery (*p* < 0.001). A significant estimated difference in fibulin-1 concentrations was identified between women with and without PPROM (-11.6 µg/mL; standard error (SE) 4.0; *p* = 0.006), where women with PPROM presented the lowest levels. A difference in fibulin-1 concentrations was not observed between women with and without PE (-0.2 µg/mL; SE 3.0; *p* = 0.9). Smokers had lower measured and predicted fibulin-1 concentrations than non-smokers (-5.2 µg/mL; SE 2.3; *p* = 0.02). In our main analysis, age, parity, BMI, mode of conception, bleeding at delivery, and birth weight did not correlate with fibulin-1 concentrations (Table 2). Additionally, in the late 3rd trimester and at delivery, we did not observe a correlation between fibulin-1 concentrations and bleeding at delivery or birth weight. On the other hand, women giving birth vaginally had higher estimated fibulin-1 concentrations than women who delivered by elective (-7.0 µg/mL; SE 2.3; *p* = 0.002) and emergency (-5.6 µg/mL; SE 2.9; *p* = 0.05) cesarean section (Table 2). In the late 3rd trimester, the mode of delivery was not correlated with fibulin-1 concentrations. After delivery, significantly lower fibulin-1 concentrations were detected in women who delivered by cesarean section, both elective (unadjusted, -13.2 µg/mL; SE 4.9; *p* = 0.008; adjusted, -14.0 µg/mL; SE 5.4; *p* = 0.01) and emergency (unadjusted, -11.2 µg/mL; SE 4.5; *p* = 0.01; adjusted, -9.1 µg/mL; SE 5.4; *p* = 0.09), than in women with vaginal delivery (reference group). See Fig. 3; Table 3 and supplementary material Figure S1 and Table S1.

**Table 2:** Estimated differences in plasma fibulin-1 concentrations between various subgroups of participants

Parameter		β-estimate (μg/mL)^**a**^	SE ^**b**^	***p***-value ^**c**^
**Intercept**		31.9	11.2	0.004 **
**Age**	Per 1-year increase	0.2	0.3	0.4
**Parity**	0	Reference		
	1	2.6	2.2	0.2
	2+	4.9	3.2	0.1
**Body mass index**	Per 1-unit increase (kg/m^2^)	-0.3	0.3	0.3
**Smoking habits**	Non-smoker	Reference		
	Smoker	-5.2	2.3	0.02 *
**Mode of conception**	Spontaneous	Reference		
	In-vitro fertilization	0.4	10.3	1.0
	Insemination	-18.1	9.5	0.06
**Gestational weeks**	12-20	Reference		
	21-28	0.9	1.4	0.5
	29-34	5.6	1.7	<0.001 ***
	35-42	12.5	1.6	<0.001 ***
	Delivery	4.1	1.5	0.005 **
	1^st^ day after delivery	-1.3	1.5	0.4
	2^nd^ day after delivery	-3.4	1.5	0.002 *
**Pre-eclampsia**	Not present	Reference		
	Present	-0.2	3.0	0.9
**PPROM**	Not present	Reference		
	Present	-11.6	4.2	0.006 **
**Mode of delivery**	Vaginal birth	Reference		
	Elective caesarean section	-7.0	2.3	0.002 **
	Emergency caesarean section	-5.6	2.9	0.05 #
**Bleeding at delivery**	Per 100mL increase	-0.03	0.4	0.9
**Birth weight**	Per 100grams increase	-0.08	0.2	0.7

^a^ Estimated slope coefficient using a linear mixed model and fitted with the restricted maximum likelihood approach. Tukey’s test was used for multiple comparisons of means. ^b^ Standard error. ^c^ Compared to the reference level of the variable, adjusted for all other variables in the table. Significance levels ‘***’0.001, ‘**’0.01, ‘*’0.05, ‘#’ 0.1

**Table 3:** Fibulin-1 levels and mode of delivery

	Unadjusted			Adjusted^**a**^		
	β-estimate (μg/mL)^**b**^	SE^**c**^	***p***-value^**d**^	β-estimate (μg/mL)^**b**^	SE^**c**^	***p***-value ^**d**^
Vaginal birth	Reference			Reference		
Elective caesarean section	-13.2	4.9	0.008 **	-14.0	5.4	0.01 *
Emergency caesarean section	-11.2	4.5	0.01 *	-9.1	5.4	0.09 **.**
*(Intercept)*	*32.9*	*1.6*	<0.001 ***	*45.3*	*19.7*	0.02 *

^a^ Adjusted for all other variables in the main regression analysis. ^b^ Estimated slope coefficient of fibulin-1 at birth, in groups stratified by mode of delivery. ^c^ Standard error. ^d^ Compared to vaginal delivery. Significance levels ‘***’0.001, ‘**’0.01, ‘*’0.05, ‘**#**’ 0.1

## Discussion

The role of fibulin-1 in the developing pregnancy is by far fully understood. In this study, we examined fibulin-1 levels longitudinally in normal pregnancy. In addition, we explored potential factors influencing variability of fibulin-1 in maternal plasma.

Plasma fibulin-1 concentrations increased significantly during pregnancy and normalized quickly after birth in women with uncomplicated pregnancies. Fibulin-1 expression in the endometrium is regulated by progesterone in a cycle-dependent manner [21, 22], and its expression is upregulated 2.8-fold in the myometrium at term [23]. High expression rates of fibulin-1 mRNA is found in the placenta, and fibulin-1 is already detected in human embryos at the 4th gestational week. The increase in plasma fibulin-1 concentration with gestational age probably reflect the high expression rates in the growing uterus, placenta, and the fetus itself. A potential explanation for the rapid decrease in plasma fibulin-1 levels observed after delivery is the elimination of circulating fibulin-1 by hemostatic processes during delivery or because the placenta and fetus are expelled [24, 25].

Quite interestingly, women who underwent elective and emergency cesarean section presented a more rapid decrease in fibulin-1 levels after birth compared to women with vaginal delivery. A plausible explanation would be that more fibulin-1 is consumed in hemostatic processes after surgical trauma, by binding to fibrinogen and mediating platelet adhesion [24, 25]. We anticipated that low levels of fibulin-1 were associated with a higher degree of bleeding at delivery. Our regression model did not support this hypothesis, potentially due to collinearity with the mode of delivery.

In a study by Liu et al., higher levels of fibulin-1 were observed in pooled serum samples collected late in the 3rd trimester from five women with severe PE than in five healthy, gestational age-matched controls [9]. However, in the present study, no difference in plasma fibulin-1 levels could be observed between the 12 women who developed PE and women with uncomplicated pregnancies. The pathophysiological mechanisms involved in PE are unclear, but early and late PE appear to be different entities. Early PE is thought to result from deficient placentation, which induces extracellular matrix remodeling, among other processes. Late PE involves a complex interaction between stressed syncytiotrophoblasts and a susceptible maternal cardiovascular system [26, 27]. In our study, no women had early PE, and few developed severe PE. This difference might explain why we did not observe a correlation between the development of PE and fibulin-1 plasma concentrations.

In 40 % of preterm deliveries, rupture of membranes is the sentinel event [11]. The recurrence rate in a subsequent pregnancy is increased by 20-fold compared to women with no previous history of PPROM [28]. Rupture of the fetal membranes is precipitated by stretch forces acting upon biochemically mediated, pre-weakened areas overlying the cervix [11]. Fibulins are structural proteins that contribute to the elastic properties of connective tissue fibers [2]. Fibulin-1 is found to be down regulated in the para-cervical weak zone, prior to rupture, being consistent with decreased rupture strength [10]. Our nine women with PPROM presented significantly lower plasma fibulin-1 concentrations throughout their pregnancies. This lack of increase in plasma fibulin-1 and the known high recurrence rate of PPROM is suggestive of a genetic explanation. Larger scale prospective studies, including Mendelian randomization, are needed to evaluate fibulin-1 as a potential prognostic biomarker for the risk of PPROM.

The two cases of women with assisted pregnancies and low fibulin-1 levels indicate that the protein might have a role in fecundity. Fibulin-1 expression is regulated by progesterone. Progesterone is a key factor in the establishing and maintaining pregnancy, and inadequate levels of postovulatory progesterone are associated with infertility and recurrent miscarriage [29]. Fibulin-1 has fibronectin-and integrin-binding properties and a role in angiogenesis. It may therefore be important for both the architecture of the endometrium and the periodic neovascularization, and as such have a functional role in endometrial receptivity for embryo implantation [21]. It would be interesting to follow women referred to fertility treatment and measure fibulin-1 before, during and after treatment.

Our study has some strengths and limitations. To our knowledge, this study is the first to date to perform a longitudinal investigation of plasma fibulin-1 levels in pregnant women. The cohort consisted of healthy women, and data on pregnancy outcomes were available. However, the study is limited by its size and the lack of pre-conceptual and first trimester samples. The few women with PE (*n* = 12) and PPROM (*n* = 9), clearly hampers any definite conclusions regarding these subgroups. All participants were Caucasian, and predominantly normal- or slightly overweight, limiting the generalizability. A comparison group consisting of women, during subsequent stages of the menstrual cycle, would have been of value. Finally, our study design did not allow us to evaluate the fibulin-1 status and its importance in fetal development.

## Conclusions

Plasma fibulin-1 levels increase significantly during pregnancy and normalize quickly after birth in women with uncomplicated pregnancies, more pronounced after elective and emergency cesarean section than after vaginal delivery. The women with late PE had fibulin-1 levels equivalent to women with uncomplicated pregnancies in our cohort. Although limited by sample size, this increase in fibulin-1 levels was not observed in pregnancies complicated with PPROM. Larger scale and validation studies are needed to determine whether fibulin-1 represents a potential prognostic biomarker for the risks of developing PPROM and PE.

## Supplementary Information

**Additional file 1.**

**Additional file 2.**

## Data Availability

The datasets used and/or analyzed during the current study are available from the corresponding author on reasonable request.

## Abbreviations

BMI: Body mass index
SE: Standard error
SD: Standard deviation
PE: Preeclampsia
PPROM: Preterm premature rupture of membranes
