# Supplementary material for: Plasma fibulin-1 levels during pregnancy and delivery: a longitudinal observational study
Source: BMC Pregnancy Childbirth. 2021 Sep 17;21:629. doi: 10.1186/s12884-021-04110-y (PMC8447534; doi:10.1186/s12884-021-04110-y)

**Plasma fibulin-1 levels during pregnancy and delivery: a longitudinal observational study**

by

Astrid Bakke Orvik, Malene Rohr Andersen, Lise Pedersen, Christian Ritz, Steen Stender, Pal Bela Szecsi

**Supporting Information**

**Figure S1. Fibulin-1 levels and mode of delivery.**

Plasma fibulin-1 concentrations in 3^rd^ trimester and around delivery measured in participants grouped according to the mode of delivery. The box plots represent the range of data from the 25^th^ to the 75^th^ percentiles, while the bar in the middle of each box plot represents the median value. The whiskers extending from the box represent the range of values excluding outliers. Circles indicate outliers.


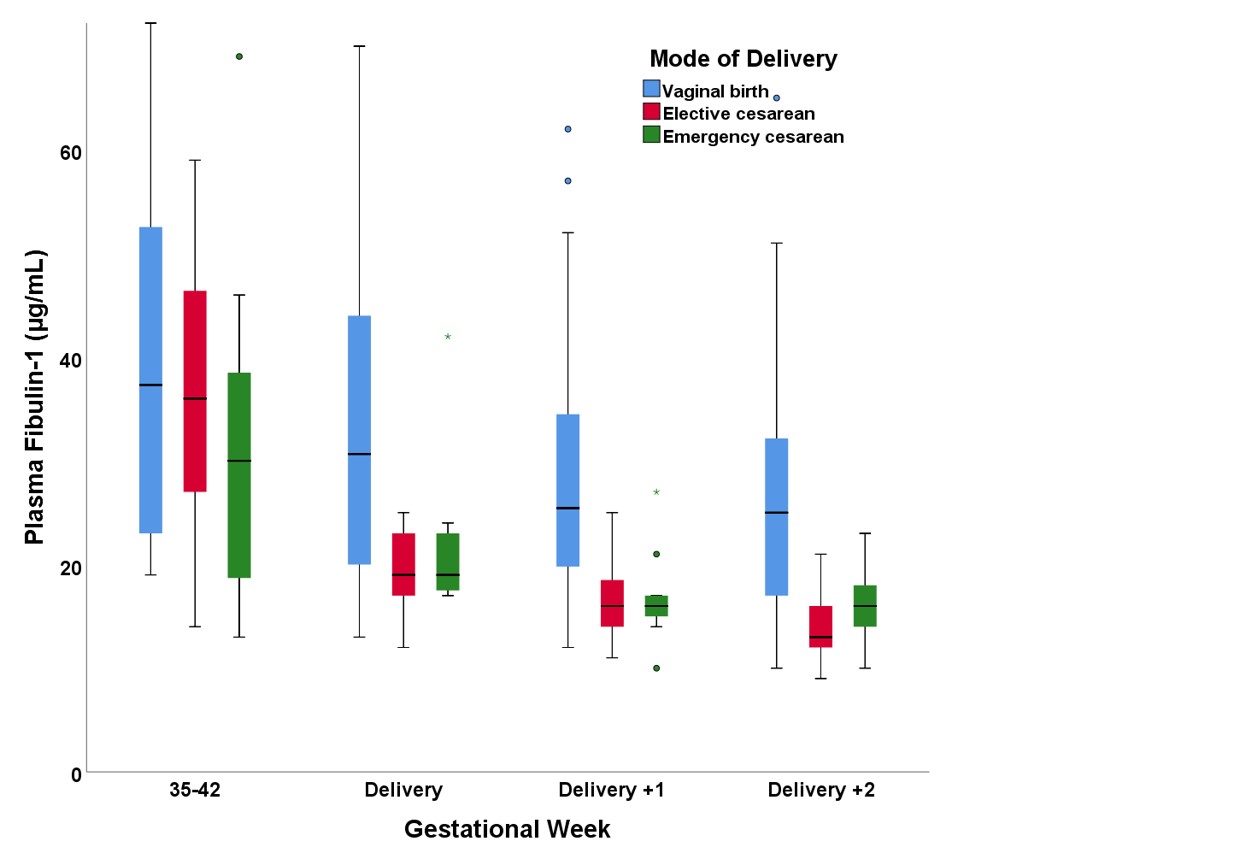

Supplement: Supplementary file 1 — Additional file 1. [file 12884_2021_4110_MOESM1_ESM.docx]
