# Supplementary material for: Plasma fibulin-1 levels during pregnancy and delivery: a longitudinal observational study
Source: BMC Pregnancy Childbirth. 2021 Sep 17;21:629. doi: 10.1186/s12884-021-04110-y (PMC8447534; doi:10.1186/s12884-021-04110-y)
Supplement: Supplementary file 2 — Additional file 2. [file 12884_2021_4110_MOESM2_ESM.docx]

**Plasma fibulin-1 levels during pregnancy and delivery: a longitudinal observational study**

by

Astrid Bakke Orvik, Malene Rohr Andersen, Lise Pedersen, Christian Ritz, Steen Stender, Pal Bela Szecsi

**Supporting Information**

**Table S1. Fibulin-1 levels and mode of delivery.**

|  | **Unadjusted** | | | **Adjusted ^a^** | | |
| --- | --- | --- | --- | --- | --- | --- |
|  | **β-estimate (µg/mL)^b^** | **SE ^c^** | **p-value ^d^** | **β-estimate (µg/mL) ^b^** | **SE ^c^** | **p-value ^d^** |
| Vaginal birth | Reference |  |  | Reference |  |  |
| Elective caesarean section | -13.2 | 4.9 | 0.008 ** | -14.0 | 5.4 | 0.01 * |
| Emergency caesarean section | -11.2 | 4.5 | 0.01 * | -9.1 | 5.4 | 0.09 # |
| *(Intercept)* | *32.9* | *1.6* | *<0.001 **** | *45.3* | *19.7* | *0.02 ** |

**^a^** Adjusted for all other variables in the main regression analysis. ^b^ Estimated slope coefficient of fibulin-1 at birth, in groups stratified by mode of delivery. ^c^ Standard error. ^d^ Compared to vaginal delivery. Significance levels ‘***’0.001, ‘**’0.01, ‘*’0.05, ‘#’ 0.1
